# Primary care in the time of COVID-19: monitoring the effect of the pandemic and the lockdown measures on 34 quality of care indicators calculated for 288 primary care practices covering about 6 million people in Catalonia

**DOI:** 10.1186/s12875-020-01278-8

**Published:** 2020-10-10

**Authors:** Ermengol Coma, Núria Mora, Leonardo Méndez, Mència Benítez, Eduardo Hermosilla, Mireia Fàbregas, Francesc Fina, Albert Mercadé, Souhel Flayeh, Carolina Guiriguet, Elisabet Balló, Nuria Martinez Leon, Ariadna Mas, Sílvia Cordomí, Yolanda Lejardi, Manuel Medina

**Affiliations:** 1 Sistemes d’Informació dels Serveis d’Atenció Primària (SISAP), Institut Català de la Salut (ICS), Gran Via de les Corts Catalanes, 587, 08007 Barcelona, Spain; 2 Fundació Institut Universitari per a la recerca a l’Atenció Primària de Salut Jordi Gol i Gurina (IDIAPJGol), Barcelona, Spain; 3 Equip d’Atenció Primària Gòtic, Institut Català de la Salut, Barcelona, Spain; 4 Equip d’Atenció Primària Gran Sol, Institut Català de la Salut, Badalona, Spain; 5 Equip d’Atenció Primària de Salt, Institut Català de la Salut, Girona, Spain; 6 Direcció Assistencial Atenció Primària, Institut Català de la Salut, Barcelona, Spain

**Keywords:** Quality Indicators, Health Care, COVID-19 [Supplementary Concept]., Chronic disease., Quaternary Prevention., Quality Assurance, Health Care., Primary health care.

## Abstract

**Background:**

To analyse the impact of the COVID-19 epidemic and the lockdown measures on the follow-up and control of chronic diseases in primary care.

**Methods:**

Retrospective study in 288 primary care practices (PCP) of the Catalan Institute of Health. We analysed the results of 34 indicators of the Healthcare quality standard (EQA), comprising different types: treatment (4), follow-up (5), control (10), screening (7), vaccinations (4) and quaternary prevention (4). For each PCP, we calculated each indicator’s percentage of change in February, March and April 2020 respective to the results of the previous month; and used the T-Student test for paired data to compare them with the percentage of change in the same month of the previous year. We defined indicators with a negative effect those with a greater negative change or a lesser positive change in 2020 in comparison to 2019; and indicators with a positive effect those with a greater positive change or a lesser negative change.

**Results:**

We observed a negative effect on 85% of the EQA indicators in March and 68% in April. 90% of the control indicators had a negative effect, highlighting the control of LDL cholesterol with a reduction of − 2.69% (95%CI − 3.17% to − 2.23%) in March and − 3.41% (95%CI − 3.82% to − 3.01%) in April; and the control of blood pressure with a reduction of − 2.13% (95%CI − 2.34% to − 1.9%) and − 2.59% (95%CI − 2.8% to − 2.37%). The indicators with the greatest negative effect were those of screening, such as the indicator of diabetic foot screening with a negative effect of − 2.86% (95%CI − 3.33% to − 2.39%) and − 4.13% (95%CI − 4.55% to − 3.71%) in March and April, respectively. Only one vaccination indicator, adult Measles-Mumps-Rubella vaccine, had a negative effect in both months. Finally, among the indicators of quaternary prevention, we observed negative effects in March and April although in that case a lower inadequacy that means better clinical outcome.

**Conclusions:**

The COVID-19 epidemic and the lockdown measures have significantly reduced the results of the follow-up, control, screening and vaccination indicators for patients in primary care. On the other hand, the indicators for quaternary prevention have been strengthened and their results have improved.

## Background

COVID-19 began as an outbreak in Wuhan, China, in December 2019 and quickly evolved into a global pandemic [1]. The first cases in Europe were confirmed in France on 24 January 2020 [2] and the first in Spain on 31 January 2020. Due to the fast spread of the disease and its health consequences, most countries established a lockdown strategy and distancing measures [3]. In Spain, these measures were adopted on 14 March 2020 [4].

Very few studies have analysed the health consequences of social isolation measures and most of them have focused on the impact on mental illness [5, 6]. Two studies have examined the effect on the control of diabetes mellitus, although the results are inconsistent [7, 8], and a few short articles suggest that these measures have reduced screening [9] and childhood vaccination [10]. However, until now, the impact of the COVID-19 epidemic and its control measures on the follow-up and control of chronic diseases, adult vaccination coverage or quaternary prevention have not been yet analysed in depth.

The health care quality indicators have been used in recent years to quantify improvements in the follow-up and control of different chronic diseases in primary care, reducing the variability of performance among primary care groups [11]. In Catalonia, the Healthcare quality standard (EQA, the Catalan acronym for *Estàndard de Qualitat Assistencial*) has been calculated for more than 10 years. The EQA is a synthetic indicator with more than 60 clinical indicators on quaternary prevention, preventive activities, and follow-up, control and treatment of different chronic diseases [12]. These indicators are considered a useful tool for measuring clinical practice and their results have been validated by more than 6000 professionals who use them monthly [13].

Our study aims to analyse the impact of the COVID-19 pandemic and lockdown measures on the results of health care quality indicators and, consequently, on the control of chronic diseases seen in primary care.

## Methods

Retrospective descriptive study conducted in the 288 primary care practices (PCP) of the *Institut Català de la Salut* (Catalan Institute of Health, ICS, for its Catalan initials). The ICS is the main provider of health services in Catalonia and its PCPs cover about 6 million people (approximately 80% of the Catalan population). Its population is highly representative of the population of Catalonia in terms of geographic area, age distribution and gender [14].

The period of the study included the results of the EQA indicators for the first 4 months of the years 2019 and 2020.

The main variable of the study was the result of the different indicators of EQA for the primary care practices. The EQA is a synthetic indicator composed of more than 60 clinical indicators. These indicators were defined through the proposals of more than 100 professionals and underwent a peer review before being included in the EQA, as established in its construction methodology [12]. The EQA was first designed in 2006 but has evolved over the years and adapted to scientific evidence. It is currently calculated in an aggregate form for all PCPs and health professionals at ICS, using data from the electronic medical records of all patients over 14 years old. In this study, data used were only the aggregated results of the indicators routinely calculated in our health system. We didn’t perform any data extraction or analysis of patient’s information.

For this study, 34 indicators of different types were included: adequacy of treatment (4 indicators), follow-up of chronic diseases (5), control of chronic diseases (10), screening (7), vaccinations (4) and quaternary prevention (4). We selected those EQA indicators more related to systematic activities of primary care, chronic conditions and vaccination and we excluded indicators about acute diseases or without data available for the study period, such as influenza vaccination indicators. The full list, definition and short name of these indicators is detailed in Additional file 1.

The indicators were analysed globally and according to rurality and socio-economic status. Rural areas were defined as areas with less than 10,000 inhabitants and a population density lower than 150 inhabitants/km2. We assessed the socioeconomic status of the PCP using the validated MEDEA deprivation index [15], calculated on the basis of census sections in urban areas and aggregated by calculating the patients weighted average of the sections covered by a PCP. This aggregation is routinely done by ICS in order to assign a socioeconomic status to each PCP. We categorised MEDEA deprivation index into quartiles where 1st and 4th quartiles are least and most deprived areas, respectively. Rural areas were categorised separately.

### Statistical analysis

For each PCP, we calculated the percentage of change in the results of the 40 indicators analysed compared to the previous month. We define the percentage of change as $\left(\frac{{resul t}_t}{resul_{t-1}}-1\right)\times 100$, where *t* is the corresponding month of study.

Then, for each month, we calculated the difference between the percentage of change of 2020 and 2019 and applied the T-Student test for paired data. We considered that there were significant differences between 2019 and 2020 when the *p*-value was lower than 0.05.

We defined indicators with a negative effect as those indicators with a greater negative change or a lesser positive change in the months of 2020 compared to those of 2019. We defined indicators with a positive effect as those with a smaller negative change or a larger positive change in the months of 2020 compared to 2019.

Finally, we calculated the percentage of indicators with negative effect and positive effect for each group.

All analyses have been conducted using R, version 3.5.1 [16].

## Results

Table 1 summarizes the characteristics of the PCP included in our study. 66% of the PCP analyzed were urban and close to 20% were deprived areas.

**Table 1:** Demographic features of PCP included in the study

Variable	Mean	SD	Minimum	25th percentile	Median	75th percentile	Maximum
Mean age of patients (years)	49	1.9	42	47.7	49	50	55.3
Population assigned to practices	17,171.7	7592.5	2018	11,976	17,412	22,544	40,131
% of women	50.8	2	39.5	49.7	50.8	51.9	57.8
% of immigration from a low-income country	12.9	7	2.6	7.9	11.4	15.7	40.3
**Variable**	**Value**	**N**	**%**				
Socioeconomic status (MEDEA)	Urban - Quartile 1 (Least deprived)	48	16.7				
Socioeconomic status (MEDEA)	Urban - Quartile 2	36	12.5				
Socioeconomic status (MEDEA)	Urban - Quartile 3	51	17.1				
Socioeconomic status (MEDEA)	Urban - Quartile 4 (Most deprived)	54	18.8				
Rurality	Rural	99	34.4				

The indicators with statistically significant negative and positive effect per month are shown in Table 2. Eighty-five percent of the EQA indicators studied had a negative effect in the month of March and 68% also had a negative effect in the month of April. In contrast, in the month of February only 4 indicators (12%) showed this negative effect. Conversely, the positive effects were greater in the month of February with 10 indicators (29.4%) having this effect but 0 in March and only 1 (2.9%) in April.

**Table 2:** Number and percentage of indicators with statistically significant negative and positive effect per month

Indicator type	Effect	February	March	April
Vaccinations	–		2 (50%)	2 (50%)
	+	3 (75%)		
Screening	–	2 (28.57%)	5 (71.43%)	5 (71.43%)
	+			
Follow-up	–	2 (40%)	5 (100%)	4 (80%)
	+			
Treatment	–		4 (100%)	
	+	2 (50%)		
Control	–		9 (90%)	9 (90%)
	+	2 (20%)		
Quaternary prevention	–		4 (100%)	3 (75%)
	+	3 (75%)		1 (25%)
**Total**	**–**	**4** **(11.8%)**	**29** **(85.3%)**	**23** **(67.7%)**
	**+**	**10** **(29.4%)**		**1** **(2.9%)**

Legend: Cells are left blank when no indicator had a negative (−) or positive (+) effect.

All the effects presented are statistically significant. The exact *p*-value and the confidence intervals of the differences can be found in the Additional file 2.

**Fig. 1:**
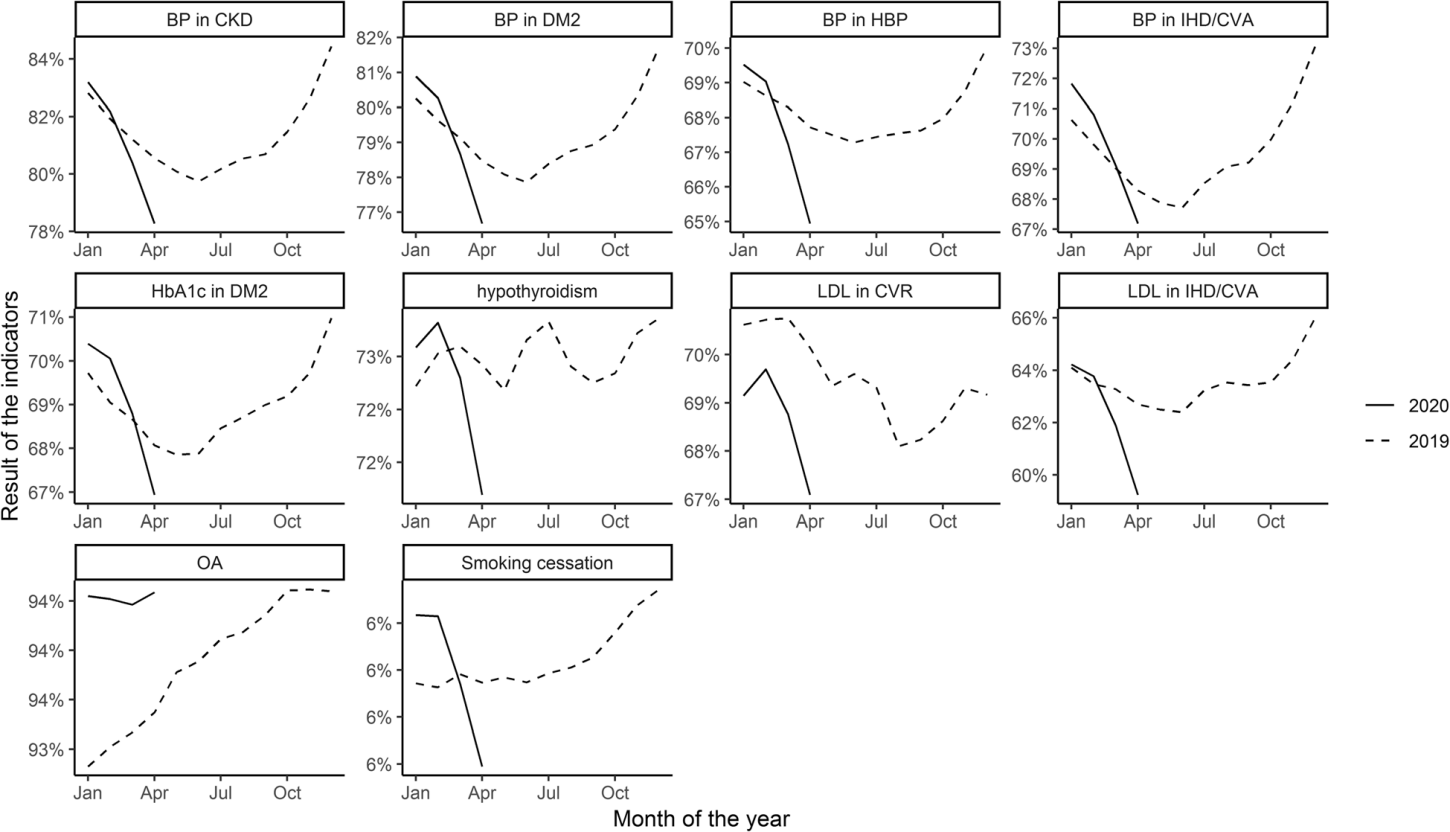
Monthly result of EQA control indicators during 2019 and 2020. Legend: Short name of each indicator can be found in Additional file 1

9 out of 10 control indicators (90%) had a negative effect in the months of March and April. Figure 1 shows the evolution of the monthly result of these indicators.

At the level of each indicator, the LDL control indicator in IHD/CVA patients decreased the result by − 2.9% and − 4.3% in March and April 2020, respectively. This reduction was significantly higher than that of the same months of the previous year which was − 0.23% and − 0.89%; overall difference of − 2.69% (95% CI − 3.17% to − 2.23%, *p*-value< 0.05) in March and − 3.41% (95% CI − 3.82% to − 3.01%, *p*-value< 0.05) in April. Similarly, the glycated haemoglobin A (HbA1c) control indicator in type 2 diabetes mellitus decreased by − 1.2% (95% CI − 1.42% to − 0.99%, *p*-value< 0.05) more in March 2020 and − 1.86% (95% CI − 2.06% to − 1.65%, *p*-value< 0.05) more in April 2020 compared to the same months in 2019; and the blood pressure control indicator decreased the result by − 2.13% (95% CI − 2.34% to − 1.9%, *p*-value< 0.05) and − 2.59% (95% CI − 2.8% to − 2.37%, *p*-value< 0.05) more in March and April 2020 compared to 2019. However, the result of the indicator of accurate control of anticoagulants in atrial fibrillation was not affected during these months. The changes in each indicator during the months of February, March and April 2019 and 2020 and their differences are shown in the Additional file 2.

**Fig. 2:**
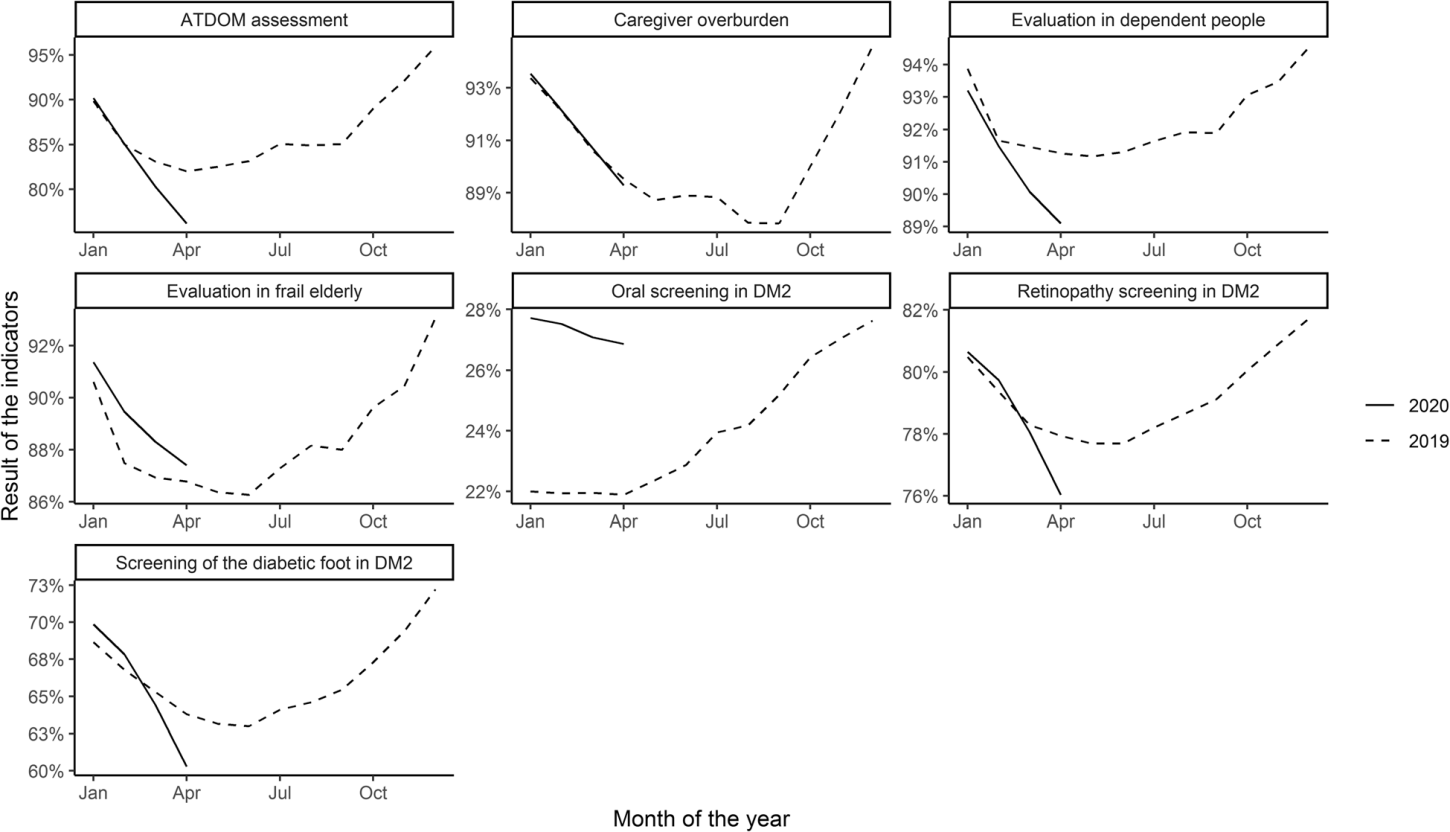
Monthly result of EQA screening indicators during 2019 and 2020. Legend: Short name of each indicator can be found in Additional file 1

Five out of seven screening indicators had a statistically significant negative effect in March and April 2020 (Table 2). Figure 2 shows the evolution of this group of indicators.

The indicators with the greatest negative effect in this group were the indicator of diabetic foot screening with a − 2.86% (95% CI − 3.33% to − 2.39%; *p*-value< 0.05) and a − 4.13% (95% CI − 4.55% to − 3.71%; *p-*value< 0.05) further reduction in March and April 2020 compared to 2019; the indicator of comprehensive assessment in people with home care with a − 3.39% (95% CI − 4.29% to − 2.5%; *p-*value< 0.05) further reduction in March and a − 4.01% (95% CI − 4.94% to − 3.09%, *p-*value< 0.05) further reduction in April; and the indicator of retinopathy screening in type 2 diabetes mellitus with a difference in percentages of change between 2019 and 2020 of − 0.78% (95% CI − 1.14% to − 0.44%, *p-*value< 0.05) in March and − 2.18% (95% CI − 2.46% to − 1.91%, *p-*value< 0.05) in April. The remaining indicators and their differences can be found in Additional file 2.

Quaternary prevention indicators had both negative and positive effects. All four indicators had a negative effect in March and three of them (75%) had the effect also in April. This is the case of the indicator of new inadequately prescribed statins that decreased by − 2.28% in March 2020 and by − 3.85% in April 2020 in comparison with the 1.05 and 0.05% change in March and April 2019, respectively. The Incorrect use of prostatic-specific antigen (PSA) indicator was reduced by a − 4.45% (95% CI − 6.16% to − 2.7%; *p*-value< 0.05) more in March 2020 and a − 4.73% (95% CI − 6.23% to − 3.23%; *p-*value< 0.05) more in April 2020 than in the same months of the previous year (see Additional file 2).

The 4 treatment indicators had a negative effect in March but none in April (Table 2). In addition, half of the vaccination indicators had a negative effect, although only one had a negative effect for two consecutive months: the Measles-Mumps-Rubella (MMR) vaccination indicator. This negative effect was lower than in other indicators, with a decrease of − 0.22% (95% CI − 0.32% to − 0.12%; *p-*value< 0.05) and − 0.14% (95% CI − 0.25% to − 0.04%; *p-*value< 0.05) more in the months of March and April 2020, respectively (see Table 2 and Additional file 2).

The number of patients with type 2 diabetes mellitus, hypertension and atrial fibrillation taken care of and the number of patients treated with anticoagulants with 6 or more controls in primary care were significantly reduced. In March 2020, these follow-up indicators decreased, compared to 2019, by − 0.51% (95% CI − 0.6% to − 0.42%, *p*-value< 0.05), − 0.48% (95% CI − 0.56% to − 0.39%; *p-*value< 0.05), − 0.67% (95% CI − 0.94% to − 0.38%; *p-*value< 0.05) and − 1.08% (95% CI − 1.59% to − 0.59%; *p-*value< 0.05), respectively (see Additional file 2).

**Fig. 3:**
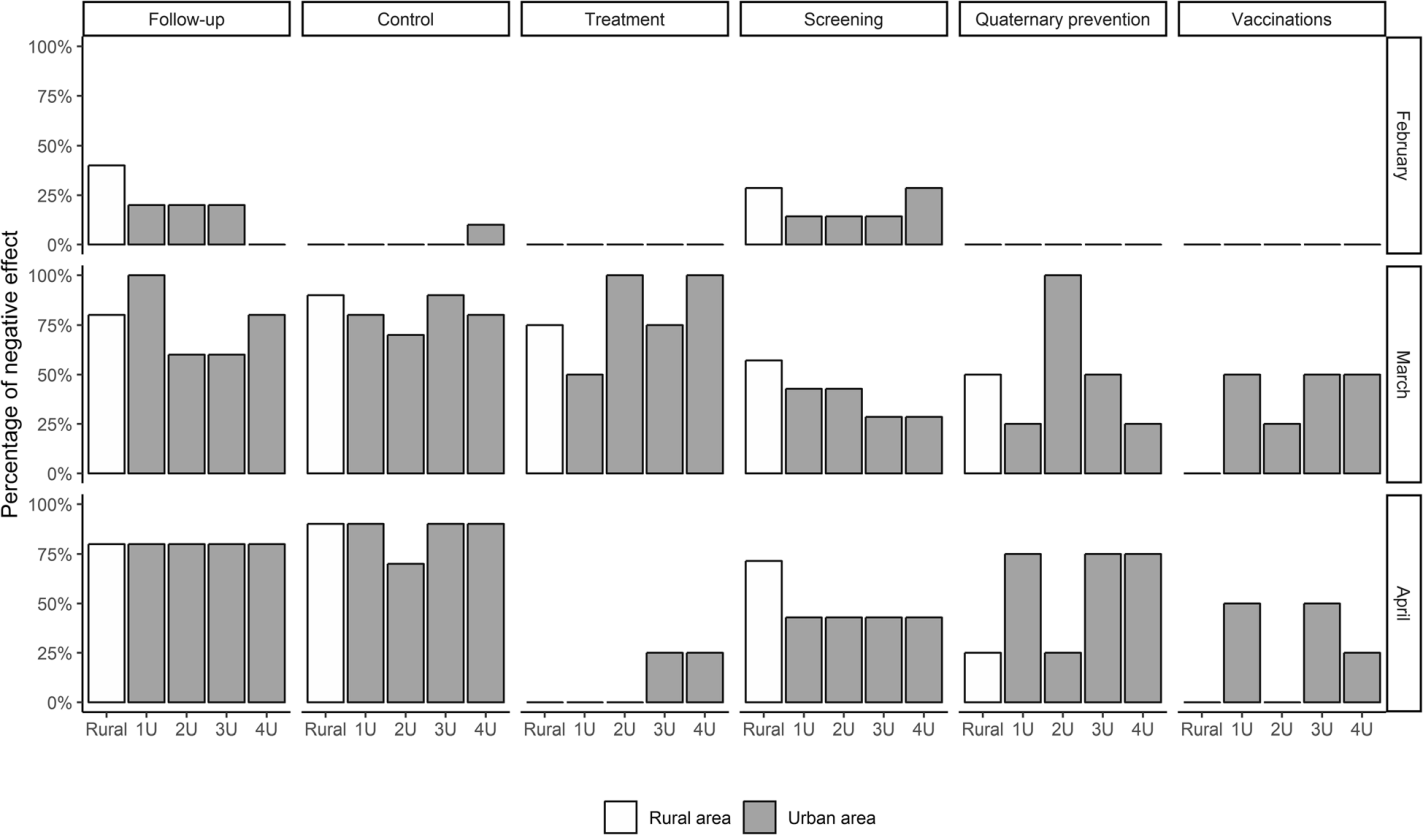
Percentage of indicators with significant negative effect according to rurality and socioeconomic status in urban areas. Legend: socioeconomic status in urban areas: 1 U - first quartile urban least deprived; 2 U - second quartile; 3 U - third quartile; 4 U - fourth quartile

Figure 3 summarises the positive and negative effects according to rurality and the socioeconomic status of the PCPs. Overall, rural PCPs had less negative effect on vaccination indicators with 0% of the indicators with negative effect in March and April versus 50% (2/4) in urban PCPs; and on quaternary prevention indicators with 25% (1/4) of the indicators with negative effect in March versus 75% (3/4) in urban PCPs. In addition, they had more negative effect on screening indicators: 57% (4/7) and 71% (5/7) of the indicators with negative effect in March and April respectively in rural PCPs versus 43% (3/7) in urban PCPs. No differences were observed in the positive and negative effects according to socioeconomic status.

Monthly results of all groups of EQA indicators are shown in Additional file 3.

## Discussion

### General overview

This is the first study to our knowledge analysing the effects of the COVID-19 epidemic and its control measures on the results of health care quality indicators in primary care. Four out of five indicators in our study had what we call a negative effect in the month of March; in other words, a greater reduction in the results when compared to the same period last year. In April, this effect appeared in more than half of the indicators. The negative effect temporarily overlapped with the lockdown measures established in Spain in mid-March. It should be noted that before these measures, there were more indicators with a positive effect (increase in the percentage of compliance when compared to last year) than with a negative effect, as reflected in the data for the month of February.

#### Follow-up, control screening and vaccination indicators

In our study, there was a decrease in follow-up indicators such as the number of type 2 diabetics taken care of, the number of hypertensive patients taken care of or the number of controls in patients with anticoagulants (although not their degree of control); in control indicators such as HbA1c control in type 2 diabetes mellitus, blood pressure control in hypertensives or LDL control in ischemic heart disease (IHD) and cerebrovascular accident (CVA) patients; in screening indicators such as diabetic foot screening or diabetic retinopathy screening; and even in vaccination indicators such as MMR vaccination in adults, although their negative variation was much slighter.

These reductions arose in the context of the exceptional situation experienced during these months in Catalonia and most countries in the world. On the one hand, due to a high incidence of COVID-19 cases (more than 120,000 cases taken care of in primary care [17]) requiring priority assistance in the health system and, on the other hand, due to the recommendations of health authorities who advised against going to health centres except in the event of serious illness or urgent situations.

Although the follow-up, screening and vaccination indicators of the EQA are based on scientifically proven actions with numerous benefits [18, 19], these are neither urgent nor vital actions which must be carried out in the short term. It is therefore understandable that they be postponed for some time and is consistent with what has happened in other countries [20, 21]. In the United States, for example, they also observed a reduction in vaccines administered to children during the same months as in our study [10], and in Italy, cervical smears decreased but not the detection of malignant neoplasms [9]. However, these delays in some activities may have future consequences, such as worse detection of diseases, a certain overloading of the system when elective procedures are resumed if these delays are prolonged in time [22], and effects on the population’s health. In a study about the impact of the lockdown’s duration on glycaemic control, it was estimated an increase in HbA1c and an increase in complications in diabetic patients proportional to the days of lockdown [8]. On the contrary, another study found no worsening of type 1 diabetes mellitus control before and after the COVID-19 lockdown measures [7]. Further studies will therefore be needed to analyse how long it takes to recover the results of the health care quality indicators and the possible health consequences of a long-term negative effect.

#### Treatment indicators

The treatment indicators deserve a separate comment. These indicators measure prescriptions issued by primary care physicians. Their negative effect in the month of March probably had no real impact on the population since during the state of alarm in Spain, patients could still pick up their medication from the pharmacy even if the prescription was expired [23]. However, as of the end of May this was no longer possible, although the telematic renewal of the prescription was available without the need to physically carry the written prescription [24].

#### Quaternary prevention indicators

In our study, the indicators of quaternary prevention had a negative effect in March and April. For example, inadequate screening of PSA or new inadequately prescribed statins indicators were significantly reduced. This situation has a positive reading: in these indicators, the negative effect means an improvement in the indicator because it means a reduction in inadequacy. Nevertheless, the overall negative effect found in almost all control, follow-up and screening indicators leads us to suspect that not only inadequate PSA screening and inadequate statin prescriptions have been reduced, but also overall PSA screening and statin prescriptions; both inadequate and necessary. This suspicion should be contrasted with specific work focusing on the effects of the COVID-19 epidemic and its control measures on the quaternary prevention.

#### Limitations and strengths

Our research has a number of limitations. Firstly, even though most of the indicators decreased, we cannot determine the direct impact on our patients’ health. Nonetheless, EQA has proven to be a good measure of our population’s health and its indicators have been used for more than a decade to measure health outcomes [12]. Consequently, a widespread negative effect such as the one observed should at least serve as a warning signal. Secondly, the very design of our study does not allow us to ensure a causal correlation between lockdown measures and the reduction of quality indicators, but only a temporal coincidence. It should be noted, though, that the negative effect was almost non-existent in the month prior to the lockdown: only four indicators had a negative effect in February and almost a third had a positive effect. These data contrasts with the subsequent months and reinforces the correlation between the COVID-19 epidemic and its control measures and the decline in health care quality indicators. Further studies must be performed (perhaps with time-series methodology) to ensure this relation if the decrease of the results of the indicators last for more time. Thirdly, within the control indicators of our study (such as the control of HbA1c in type 2 diabetes mellitus), we could not separate patients who had a control below the standard value from those who could not be controlled, as both cases are counted as not controlled. The second situation would seem to be the most common due to the general impact on the follow-up and screening indicators. However, specific studies are needed to confirm or disprove this hypothesis.

This study also has many strengths. EQA indicators have been shown to be useful in improving clinical situations [12]. Moreover, they are widely accepted among health professionals and have been used to improve feedback tools which have been proven to be very effective [25]. The EQA indicator system and its criteria are also standardised across all centres and, therefore, our conclusions are scalable across Catalonia. Finally, our research has analysed 34 health care quality indicators comprising different aspects of clinical practice which provide us with a global vision of the effects of the COVID-19 epidemic and its control measures on the care of other patients.

## Conclusions

This is the first study up to date to analyse the consequences of the COVID-19 epidemic and its control measures on the results of health care quality indicators. In our research, the follow-up, control, screening and vaccinations of patients in primary care were significantly reduced. In contrast, the indicators of quaternary prevention, of not doing, were reinforced with improvements in results. COVID-19 control measures are widespread at global scale, and although their need is clear, the impact on systematic activities of PCPs that our results show could hold in other settings with similar health systems, pandemic burden and pandemic response. And raises awareness that progress must continue to be made in other challenges not related to SARS-COV-2 infection, being essential to restore all interrupted care activity as soon as possible to minimise the health effects of an extended worsening of results.

## Supplementary information

**Additional file 1.** Definition of the health care quality indicators included in the study with their short descriptor used in the graphics of this article.

**Additional file 2.** Percentage of change, difference and significance of the 40 health care quality indicators in the months of February, March and April 2019 and 2020.

**Additional file 3: Fig. A** Monthly result of EQA treatment indicators during 2019 and 2020. **Fig. B** Monthly result of EQA follow-up indicators during 2019 and 2020. **Fig. C** Monthly result of EQA vaccination indicators during 2019 and 2020. **Fig. D** Monthly result of EQA quaternary prevention indicators during 2019 and 2020.

## Data Availability

The datasets used and/or analyzed during the current study are available from the corresponding author on reasonable request.
