# Supplementary material for: Primary care in the time of COVID-19: monitoring the effect of the pandemic and the lockdown measures on 34 quality of care indicators calculated for 288 primary care practices covering about 6 million people in Catalonia
Source: BMC Fam Pract. 2020 Oct 10;21:208. doi: 10.1186/s12875-020-01278-8 (PMC7547821; doi:10.1186/s12875-020-01278-8)
Supplement: Supplementary file 1 — Additional file 1. Definition of the health care quality indicators included in the study with their short descriptor used in the graphics of this article. [file 12875_2020_1278_MOESM1_ESM.docx]

**Additional file 1**

**Definition of the health care quality indicators included in the study with their short descriptor used in the graphics of this article**

| Indicator | Definition | Short name |
| --- | --- | --- |
| Control Indicators | | |
| Accurate control of treatment with oral anticoagulants (OA) | Percentage of people aged 14-90 years old diagnosed with atrial fibrillation and treated with oral anticoagulants (vitamin K antagonists) controlled at primary care (with 6 or more controls in primary care in the last year) with accurate control. | OA |
| LDL cholesterol control in ischemic heart diseases (IHD) and cerebrovascular accident (CVA) | Percentage of people aged 14 to 80 years old, diagnosed with TIA and/or Ischemic stroke and/or Ischemic heart disease, whose last measurement during the LDL cholesterol evaluation period is less than or equal to 120 mg/dl. If they are not specified, they are counted as incorrect. | LDL in IHD/CVA |
| Glycated haemoglobin A (HbA1c) control in type 2 diabetes mellitus (DM2) | Percentage of population over 14 years old and under 80 years old, diagnosed with type 2 diabetes mellitus (DM2), whose last measurement of glycated haemoglobin during the evaluation period is less than or equal to 8%. If they are not specified, they are counted as incorrect. | HbA1c in DM2 |
| Blood pressure control in IHD/CVA | Percentage of population aged 14-80 years old diagnosed with IHD/CVA. The mean of their last 3 BP measurements during the evaluation period is less than or equal to 150/95. If they are not specified, they are counted as incorrect. | BP in IHD/CVA |
| Blood pressure control in type 2 diabetes mellitus (DM2) | Percentage of population aged 14-80 years old diagnosed with DM2. The mean of their last 3 BP measurements during the evaluation period is less than or equal to 150/95. If they are not specified, they are counted as incorrect. | BP in DM2 |
| Blood pressure control (BP) in hypertensive patients (HBP) | Percentage of population aged 14-80 years old diagnosed with high blood pressure (HBP).The mean of their last 3 BP measurements during the evaluation period is 150/95 (or ≤ 160/95 if aged 60 years old or over). If they are not specified, they are counted as incorrect. | BP in HBP |
| LDL control in high cardiovascular risk (CVR) patients | Percentage of population aged 15-80 years old at high cardiovascular risk whose last LDL cholesterol measurement in the evaluation period is <150m/dl. If they are not specified, they are counted as incorrect. | LDL in CVR |
| Accurate control of hypothyroidism | Percentage of people aged between 14 and 80 years old, diagnosed with hypothyroidism, with accurate control of TSH (between 0.2 and 5.6 mU/L up to 70 years old; and up to 5.9 mU/L in people over 70 years old) | hypothyroidism |
| Blood pressure control (BP) in patients with chronic kidney disease (CKD) | Percentage of population aged 14-80 years old, diagnosed with high blood pressure (HBP) and chronic kidney disease (CKD). The mean of their last 3 BP measurements during the evaluation period is less than or equal to 150/95. If they are not specified, they are counted as incorrect. | BP in CKD |
| Smoking cessation | Percentage of the visited population aged between 14 and 80 years old, with smoking dependence at the beginning of the evaluation period and whose smoking cessation has been registered during this period. | Smoking cessation |
| Treatment indicators | | |
| Appropriate treatment of atrial fibrillation (AF) | Percentage of people aged between 15 and 90 years old, diagnosed with Complete Arrhythmia caused by AF with correct indication of anticoagulant treatment according to their risk of thromboembolism (CHA2DS2VASC scale) and according to the calculation of their bleeding risk using the HAS-BLED Score. | AF |
| Antiplatelet therapy in ischemic heart disease (IHD) and cerebrovascular accident (CVA) | Percentage of population over 14 years old, diagnosed with ischemic stroke and/or ischemic heart disease, which is currently treated with Antiplatelet/anticoagulant therapy. | Antiplatelet therapy in IHD/CVA |
| Beta-blocker (BB) treatment in ischemic heart disease (IHD) and heart failure (HF) | Percentage of population aged between 14 and 90 years old, diagnosed with ischemic heart disease (IHD) or heart failure (HF), which is currently treated with beta-blockers. | BB in IHD/HF |
| Treatment with ACE inhibitors or ARBs | Percentage of population visited and aged between 14 and 90 years old, diagnosed with congestive heart failure (CHF) or nephropathy/CKD with (HBP or MD2), which is currently treated with ACEi (or ARBs in case of intolerance to ACEi). | ACE/ ARBs |
| Screening Indicators | | |
| Screening of the diabetic foot in patients with type 2 diabetes mellitus (DM2) | Percentage of population over 14 and under 80 years old, diagnosed with type 2 diabetes mellitus (DM2) who have been screened for diabetic foot | Screening of the diabetic foot in DM2 |
| Retinopathy screening in patients with type 2 diabetes mellitus (DM2) | Percentage of population aged 14-80 years old, diagnosed with type 2 diabetes mellitus (DM2), who have been screened for retinopathy in the last two years | Retinopathy screening in DM2 |
| Comprehensive assessment of people in home care (ATDOM, for its Spanish initials) | Percentage of population over 14 years old, included in the ATDOM programme (for its Spanish initials - home care). Dependency (Barthel scale), cognitive status (Pfeiffer test or any other test assessing cognitive status) and social risk have been assessed, or complexity has been evaluated, at least once during the evaluation period. | ATDOM assessment |
| Overburden of the patient's caregiver in ATDOM (for its Spanish initials - home care) | Percentage of population over 14 years old included in the ATDOM (for its Spanish initials - Home care) programme having an identified caregiver. The caregiver overburden scale has been applied (score on the Zarit scale). | Caregiver overburden |
| Social evaluation in dependent people | Percentage of people over 14 years old who are dependent and who have made the social evaluation in the last two years. | Evaluation in dependent people |
| Social evaluation in frail elderly | Percentage of people with criteria of fragility to whom social evaluation has been carried out in the last two years. | Evaluation in frail elderly |
| Oral screening in patients with type 2 diabetes mellitus (DM2) and poor glycaemic control | Percentage of population between 15 and 80 years old, diagnosed with DM2 with at least one haemoglobin glycated measurement during the evaluation period being greater than 8%. An oral examination has been performed in the last two years. | Oral screening in DM2 |
| Quaternary prevention indicators | | |
| Low cardiovascular risk (CVR) with inadequately prescribed hypolipemic drugs | Percentage of the population visited and aged between 35 and 74 years old with a CVR lower than or equal to 5% and who take statins. | Low CVR |
| New inadequately prescribed statins | Population between 35 and 74 years old with new inadequate statin prescriptions in primary prevention: with a CVR (REGICOR) <10% or without a calculated CVR (REGICOR), and which at the current cut-off, they still have the statin prescribed. | New statins |
| Persistence of new inadequately prescribed statins | Population between 35 and 74 years old with persistence of new inadequate statin prescriptions in primary prevention: with a CVR (REGICOR) <10% or without a calculated CVR (REGICOR). New statins are considered to be those prescribed in the last year. | Persistence statins |
| Incorrect use of PSA in people over 69 years old | Percentage of a male population visited and aged over 69 years old. PSA was applied for during the evaluation period. | PSA |
| Follow-up indicators | | |
| Patients with atrial fibrillation taken care of | Population taken care of in the last year, aged between 15 and 90 years old with active diagnosis of Complete Arrhythmia caused by AF. | AF |
| Patients with atrial fibrillation, treatment with oral anticoagulants and 6 or more controls in primary care in the last year | People aged between 15 and 90 years old diagnosed with atrial fibrillation and being treated with oral anticoagulants (vitamin K antagonists) controlled at primary care (with 6 or more controls in primary care in the last year) | OA |
| Patients with ischemic heart disease (IHD) and/or cerebrovascular accident (CVA) taken care of | Population taken care of and aged between 14 and 90 years old, diagnosed with ischemic stroke  and/or  Population taken care of and aged between 14 and 90 years old, with open or closed diagnosis of ischemic cardiopathology. | IHD/CVA |
| Patients with type 2 diabetes mellitus taken care of | Population taken care of in the last year, aged over 14 years old and under 80 years old, with a diagnosis of type 2 diabetes mellitus. | DM2 |
| Patients with high blood pressure taken care of | Population taken care of in the last year between 14 and 80 years old, with the diagnosis of HBP. | HBP |
| *Vaccination indicators* | | |
| Pneumococcal vaccination coverage in people over 64 years old | Percentage of population taken care of and aged between 65 and 80 years old, with correct vaccination status for pneumococcus according to the current vaccination schedule (one dose in people over 64 years old). | Pneumococcal |
| Anti-tetanus vaccination coverage for those over 39 years old | Percentage of population taken care of and aged between 39 and 80 years old with correct vaccination status for tetanus and diphtheria according to the current vaccination schedule (one dose in the last 25 years) | Anti-tetanus |
| Hepatitis B (anti-HBV) and hepatitis A (anti-HAV) vaccination in patients with hepatitis C (HCV) | Population taken care of and diagnosed with chronic hepatitis C virus, being between 14 and 80 years old, and correctly immunised against hepatitis A and hepatitis B. | HBV-HAV |
| Adult MMR Vaccination | Population visited and aged ≥ 15 years old and born after 1966, vaccinated against MMR (mumps, measles and rubella). | MMR |
