# Supplementary material for: Primary care in the time of COVID-19: monitoring the effect of the pandemic and the lockdown measures on 34 quality of care indicators calculated for 288 primary care practices covering about 6 million people in Catalonia
Source: BMC Fam Pract. 2020 Oct 10;21:208. doi: 10.1186/s12875-020-01278-8 (PMC7547821; doi:10.1186/s12875-020-01278-8)
Supplement: Supplementary file 2 — Additional file 2. Percentage of change, difference and significance of the 40 health care quality indicators in the months of February, March and April 2019 and 2020. [file 12875_2020_1278_MOESM2_ESM.docx]

**Additional file 2**

**Percentage of change, difference and significance of the 40 health care quality indicators in the months of February, March and April 2019 and 2020**

| Indicators | Month | Year 2019 | Year 2020 | Difference  [95% CI]] | p-value | Significance |
| --- | --- | --- | --- | --- | --- | --- |
| Follow-up indicators |  |  |  |  |  |  |
| Patients with atrial fibrillation taken care of | February | 0.462371689 | 0.250027 | -0.21%  [-0.47% - 0.06%] | 0.125300736 |  |
|  | March | 0.56777321 | -0.09864 | -0.67%  [-0.94% - -0.38%] | 5.37E-06 | * |
|  | April | 0.561475871 | -0.8437 | -1.41%  [-1.66% - -1.15%] | 1.17E-23 | * |
| Patients with atrial fibrillation, treatment with oral anticoagulants and 6 or more controls in primary care in the last year | February | -1.158631513 | -1.23094 | -0.07%  [-0.43% - 0.29%] | 0.703686686 |  |
|  | March | -0.742440583 | -1.8269 | -1.08%  [-1.59% - -0.59%] | 2.44E-05 | * |
|  | April | -0.607390932 | -0.89151 | -0.28%  [-0.75% - 0.18%] | 0.22740132 |  |
| Patients with ischemic heart disease or cerebrovascular accident taken care of | February | 0.216366231 | 0.156788 | -0.06%  [-0.23% - 0.12%] | 0.556300327 |  |
|  | March | 0.264773684 | -0.12779 | -0.39%  [-0.58% - -0.21%] | 3.84E-05 | * |
|  | April | 0.207661746 | -0.68834 | -0.9%  [-1.05% - -0.74%] | 4.85E-24 | * |
| Patients with type 2 diabetes mellitus taken care of | February | 0.308886439 | 0.127966 | -0.18%  [-0.27% - -0.09%] | 0.000152257 | * |
|  | March | 0.329735241 | -0.18142 | -0.51%  [-0.6% - -0.42%] | 7.49E-24 | * |
|  | April | 0.326553452 | -0.59931 | -0.93%  [-1.02% - -0.83%] | 7.29E-53 | * |
| Patients with hypertension taken care of | February | 0.342757585 | 0.192877 | -0.15%  [-0.23% - -0.06%] | 0.00071285 | * |
|  | March | 0.32432408 | -0.1543 | -0.48%  [-0.56% - -0.39%] | 2.19E-25 | * |
|  | April | 0.310990346 | -0.45633 | -0.77%  [-0.85% - -0.69%] | 7.38E-53 | * |
| Control indicators |  |  |  |  |  |  |
| Accurate control of treatment with anticoagulants | February | 0.229953724 | -0.01622 | -0.25%  [-0.57% - 0.08%] | 0.133716039 |  |
|  | March | 0.190756101 | -0.0446 | -0.24%  [-0.59% - 0.13%] | 0.205473622 |  |
|  | April | 0.28680401 | 0.153496 | -0.13%  [-0.6% - 0.33%] | 0.57261863 |  |
| LDL control in IHD/CVA | February | -0.948409376 | -0.66577 | 0.28%  [-0.17% - 0.71%] | 0.228965911 |  |
|  | March | -0.226314049 | -2.91445 | -2.69%  [-3.17% - -2.23%] | 1.43E-24 | * |
|  | April | -0.897540721 | -4.31035 | -3.41%  [-3.82% - -3.01%] | 8.10E-44 | * |
| Blood pressure control in IHD/CVA | February | -1.125744868 | -1.4054 | -0.28%  [-0.65% - 0.1%] | 0.145345356 |  |
|  | March | -1.050405293 | -2.38096 | -1.33%  [-1.76% - -0.88%] | 1.10E-08 | * |
|  | April | -1.056351224 | -2.80186 | -1.75%  [-2.12% - -1.37%] | 2.16E-17 | * |
| Glycated haemoglobin A control (HbA1c) in type 2 diabetes mellitus | February | -0.946672529 | -0.48052 | 0.47%  [0.23% - 0.71%] | 0.000170953 | * |
|  | March | -0.589668906 | -1.79025 | -1.2%  [-1.42% - -0.99%] | 1.81E-23 | * |
|  | April | -0.863351465 | -2.72122 | -1.86%  [-2.06% - -1.65%] | 5.51E-48 | * |
| Blood pressure control in type 2 diabetes mellitus | February | -0.77351592 | -0.75442 | 0.02%  [-0.19% - 0.23%] | 0.863014959 |  |
|  | March | -0.635120993 | -1.99623 | -1.36%  [-1.57% - -1.12%] | 1.99E-26 | * |
|  | April | -0.819559103 | -2.54251 | -1.72%  [-1.95% - -1.5%] | 2.25E-38 | * |
| Blood pressure control in hypertensive patients | February | -0.572839861 | -0.69744 | -0.12%  [-0.34% - 0.1%] | 0.278086375 |  |
|  | March | -0.487375037 | -2.61615 | -2.13%  [-2.34% - -1.9%] | 1.28E-51 | * |
|  | April | -0.844457951 | -3.43148 | -2.59%  [-2.8% - -2.37%] | 2.21E-69 | * |
| LDL control in high cardiovascular risk (CVR) patients | February | 0.171740795 | 0.913744 | 0.74%  [0.04% - 1.45%] | 0.03756732 | * |
|  | March | 0.17127316 | -1.3053 | -1.48%  [-2.1% - -0.84%] | 6.99E-06 | * |
|  | April | -0.865575815 | -2.41652 | -1.55%  [-2.13% - -0.97%] | 2.75E-07 | * |
| Accurate control of hypothyroidism | February | 0.446644043 | 0.33438 | -0.11%  [-0.38% - 0.16%] | 0.427512049 |  |
|  | March | 0.080296268 | -0.70839 | -0.79%  [-1.11% - -0.48%] | 1.24E-06 | * |
|  | April | -0.22505632 | -1.51508 | -1.29%  [-1.55% - -1.03%] | 8.05E-20 | * |
| Blood pressure control in patients with chronic kidney disease (CKD) | February | -1.051981502 | -1.19796 | -0.15%  [-0.5% - 0.24%] | 0.476501207 |  |
|  | March | -0.8777664 | -2.16797 | -1.29%  [-1.71% - -0.86%] | 5.99E-09 | * |
|  | April | -0.757695059 | -2.64802 | -1.89%  [-2.3% - -1.48%] | 1.52E-17 | * |
| Smoking cessation | February | -0.129836489 | 0.058515 | 0.19%  [-0.8% - 1.17%] | 0.713246079 |  |
|  | March | 0.989492469 | -4.38003 | -5.37%  [-6.33% - -4.37%] | 7.07E-23 | * |
|  | April | -0.378638358 | -5.75553 | -5.38%  [-6.16% - -4.59%] | 1.62E-32 | * |
| Treatment indicators |  |  |  |  |  |  |
| Appropriate treatment of atrial fibrillation (AF) | February | -0.072349231 | -0.02421 | 0.05%  [-0.16% - 0.25%] | 0.653792437 |  |
|  | March | 0.197188982 | -0.37935 | -0.58%  [-0.78% - -0.37%] | 8.48E-08 | * |
|  | April | 0.020411972 | 0.090926 | 0.07%  [-0.12% - 0.26%] | 0.469173617 |  |
| Antiplatelet therapy in ischemic heart disease (IHD) and cerebrovascular accident (CVA) | February | -0.172360965 | -0.12908 | 0.04%  [-0.13% - 0.22%] | 0.623287515 |  |
|  | March | 0.162236401 | -0.63646 | -0.8%  [-0.98% - -0.62%] | 1.58E-16 | * |
|  | April | -0.077369609 | -0.15293 | -0.08%  [-0.24% - 0.09%] | 0.364254935 |  |
| Beta-blocker (BB) treatment in ischemic heart disease (IHD) and heart failure (HF) | February | -0.813527693 | 0.044012 | 0.86%  [0.26% - 1.43%] | 0.005075471 | * |
|  | March | -0.007868794 | -1.34144 | -1.33%  [-1.94% - -0.74%] | 1.51E-05 | * |
|  | April | 0.048869191 | 0.01284 | -0.04%  [-0.63% - 0.56%] | 0.905731667 |  |
| Treatment with ACEi or ARBs | February | -0.319917787 | -0.06181 | 0.26%  [0.05% - 0.48%] | 0.016056982 | * |
|  | March | 0.197917979 | -0.72802 | -0.93%  [-1.16% - -0.7%] | 5.82E-14 | * |
|  | April | 0.059832257 | -0.0071 | -0.07%  [-0.3% - 0.17%] | 0.571964738 |  |
| Screening Indicators |  |  |  |  |  |  |
| Screening of the diabetic foot in patients with type 2 diabetes mellitus | February | -2.681449387 | -2.88399 | -0.2%  [-0.56% - 0.17%] | 0.297437882 |  |
|  | March | -2.205730069 | -5.07054 | -2.86%  [-3.33% - -2.39%] | 5.73E-27 | * |
|  | April | -2.295436554 | -6.42699 | -4.13%  [-4.55% - -3.71%] | 1.91E-53 | * |
| Diabetic retinopathy screening in patients with type 2 diabetes mellitus | February | -1.399581913 | -1.1262 | 0.27%  [-0.04% - 0.6%] | 0.087997659 |  |
|  | March | -1.328984452 | -2.11216 | -0.78%  [-1.14% - -0.44%] | 1.28E-05 | * |
|  | April | -0.424357342 | -2.60874 | -2.18%  [-2.46% - -1.91%] | 8.33E-40 | * |
| Comprehensive assessment of people in home care (ATDOM, for its Spanish initials) | February | -5.37688301 | -5.6416 | -0.26%  [-0.92% - 0.45%] | 0.502300092 |  |
|  | March | -2.283821952 | -5.67321 | -3.39%  [-4.29% - -2.5%] | 8.59E-13 | * |
|  | April | -1.22760217 | -5.24069 | -4.01%  [-4.94% - -3.09%] | 6.69E-16 | * |
| Overburden of the patient's caregiver in ATDOM (for its Spanish initials - home care) | February | -1.344876031 | -1.47991 | -0.14%  [-0.54% - 0.25%] | 0.48134611 |  |
|  | March | -1.644638077 | -1.57075 | 0.07%  [-0.35% - 0.5%] | 0.728230393 |  |
|  | April | -1.214038075 | -1.58173 | -0.37%  [-0.8% - 0.07%] | 0.095626471 |  |
| Social evaluation in dependent people | February | -0.684935555 | -1.85861 | -1.17%  [-1.63% - -0.87%] | 4.60E-10 | * |
|  | March | 1.080000474 | -1.54276 | -2.62%  [-4.41% - -0.98%] | 0.002221688 | * |
|  | April | -0.067296344 | -1.07864 | -1.01%  [-1.73% - -0.47%] | 0.000630614 | * |
| Social evaluation in frail elderly | February | -0.838148688 | -1.91631 | -1.08%  [-1.84% - -0.8%] | 9.34E-07 | * |
|  | March | 1.872436147 | -1.2169 | -3.09%  [-6.66% - 0.16%] | 0.061747808 |  |
|  | April | 1.694923551 | -0.85346 | -2.55%  [-4.63% - -0.93%] | 0.00336939 | * |
| Oral screening in patients with type 2 diabetes mellitus and poor glycaemic control | February | 0.591510076 | -0.26157 | -0.85%  [-2.34% - 0.72%] | 0.299195902 |  |
|  | March | 1.079755861 | -1.62709 | -2.71%  [-4.45% - -1%] | 0.002062508 | * |
|  | April | -0.077365108 | -0.73446 | -0.66%  [-2.37% - 1.06%] | 0.451372958 |  |
| Quaternary prevention indicators |  |  |  |  |  |  |
| Low CVR with inadequately prescribed hypolipemic drugs | February | -0.425561791 | 0.218637 | 0.64%  [0.21% - 1.1%] | 0.004363387 | * |
|  | March | 0.276315141 | -0.88105 | -1.16%  [-1.58% - -0.73%] | 2.15E-07 | * |
|  | April | -0.552929259 | 0.280882 | 0.83%  [0.44% - 1.23%] | 4.96E-05 | * |
| New inadequately prescribed statins | February | -0.962200685 | 2.040198 | 3%  [1.27% - 4.81%] | 0.000805589 | * |
|  | March | 1.050916241 | -2.28602 | -3.34%  [-5.42% - -1.19%] | 0.002285137 | * |
|  | April | 0.054038967 | -3.8514 | -3.91%  [-5.69% - -2.12%] | 2.33E-05 | * |
| Persistence of new inadequately prescribed statins | February | 0.928325753 | 5.884345 | 4.96%  [2.31% - 7.66%] | 0.00028636 | * |
|  | March | 4.527127456 | -1.10558 | -5.63%  [-8.18% - -2.97%] | 3.43E-05 | * |
|  | April | 1.873504543 | -4.21578 | -6.09%  [-8.39% - -3.78%] | 3.79E-07 | * |
| Incorrect use of PSA in people over 69 years old | February | 1.003885482 | 2.462284 | 1.46%  [-0.05% - 2.82%] | 0.05827468 |  |
|  | March | 2.867814078 | -1.58522 | -4.45%  [-6.16% - -2.7%] | 8.08E-07 | * |
|  | April | -0.528516554 | -5.25988 | -4.73%  [-6.23% - -3.23%] | 1.92E-09 | * |
| Vaccination indicators |  |  |  |  |  |  |
| Pneumococcal vaccination coverage in people over 64 years old | February | -0.349918045 | -0.27949 | 0.07%  [0% - 0.14%] | 0.040182825 | * |
|  | March | -0.419183404 | -0.44644 | -0.03%  [-0.09% - 0.04%] | 0.419842894 |  |
|  | April | -0.371600162 | -0.50438 | -0.13%  [-0.19% - -0.08%] | 8.11E-06 | * |
| Anti-tetanus vaccination coverage for those over 39 years old | February | -0.068480203 | -0.0256 | 0.04%  [0% - 0.08%] | 0.054931456 |  |
|  | March | -0.079859745 | -0.22207 | -0.14%  [-0.19% - -0.1%] | 1.58E-10 | * |
|  | April | -0.05065299 | -0.0346 | 0.02%  [-0.02% - 0.05%] | 0.389473077 |  |
| HBV and HAV vaccination in HCV | February | -0.214647894 | 0.381955 | 0.6%  [0.07% - 1.09%] | 0.025139615 | * |
|  | March | 0.170584354 | -0.02185 | -0.19%  [-0.79% - 0.38%] | 0.50013017 |  |
|  | April | -0.046714306 | -0.27873 | -0.23%  [-0.68% - 0.22%] | 0.313774471 |  |
| Adult MMR Vaccination | February | 0.284943321 | 0.6268 | 0.34%  [0.25% - 0.43%] | 6.68E-13 | * |
|  | March | 0.449581845 | 0.234146 | -0.22%  [-0.32% - -0.12%] | 1.81E-05 | * |
|  | April | 0.46601031 | 0.326258 | -0.14%  [-0.25% - -0.04%] | 0.008402423 | * |
