# Supplementary material for: Primary care in the time of COVID-19: monitoring the effect of the pandemic and the lockdown measures on 34 quality of care indicators calculated for 288 primary care practices covering about 6 million people in Catalonia
Source: BMC Fam Pract. 2020 Oct 10;21:208. doi: 10.1186/s12875-020-01278-8 (PMC7547821; doi:10.1186/s12875-020-01278-8)
Supplement: Supplementary file 3 — Additional file 3: Fig. A Monthly result of EQA treatment indicators during 2019 and 2020. Fig. B Monthly result of EQA follow-up indicators during 2019 and 2020. Fig. C Monthly result of EQA vaccination indicators during 2019 and 2020. Fig. D Monthly result of EQA quaternary prevention indicators during 2019 and 2020. [file 12875_2020_1278_MOESM3_ESM.docx]

**Additional file 3**

**Figure A Monthly result of EQA treatment indicators during 2019 and 2020**


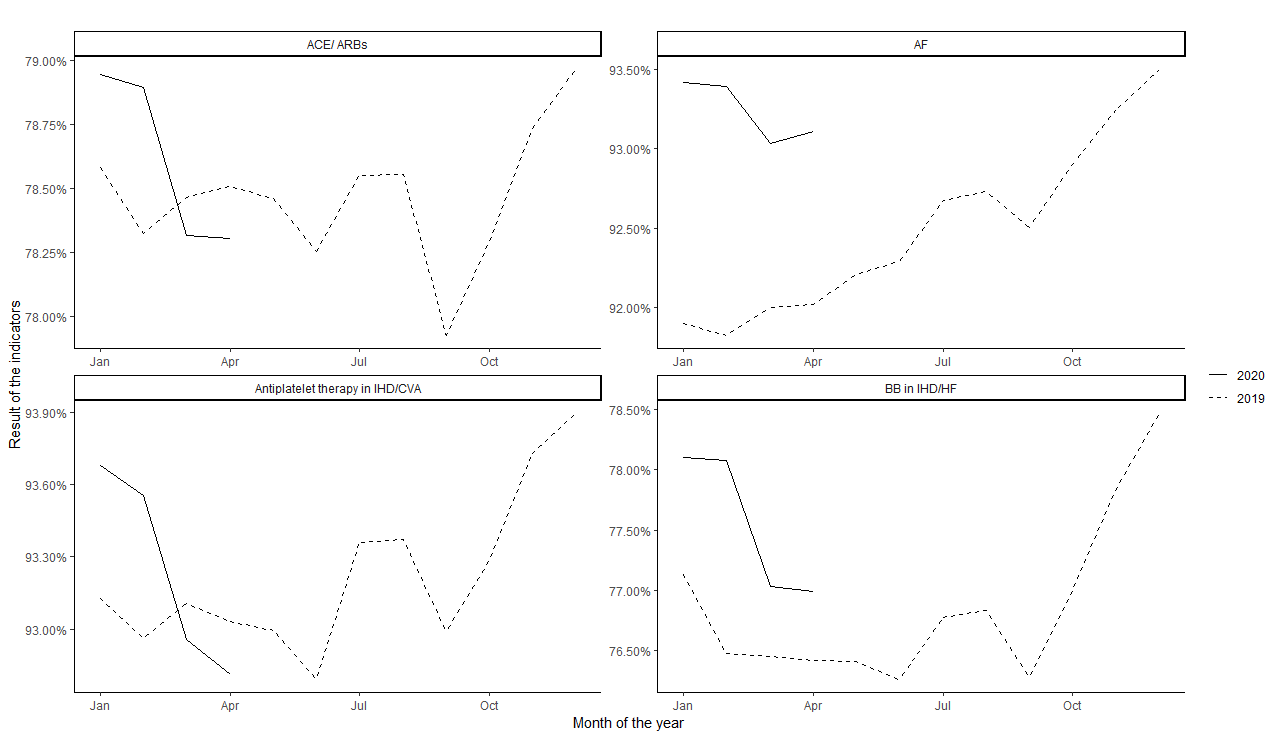


**Figure B Monthly result of EQA follow-up indicators during 2019 and 2020**


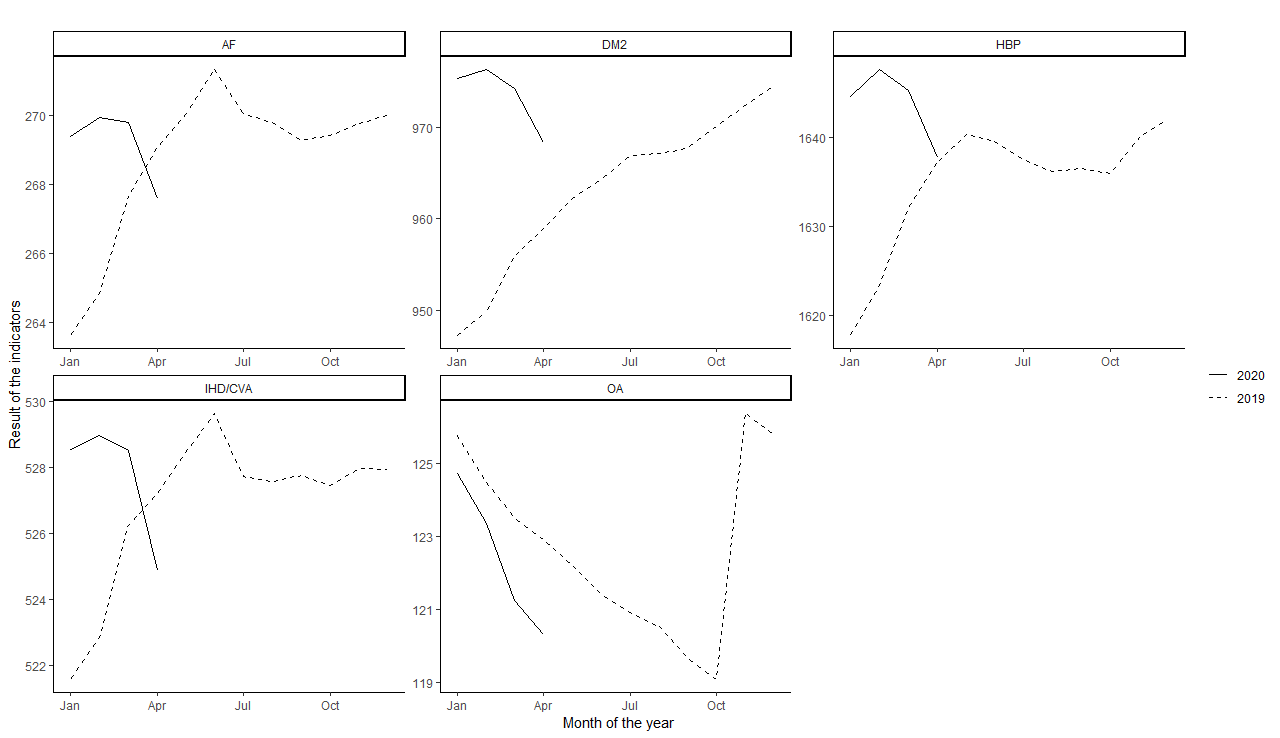


**Figure C Monthly result of EQA vaccination indicators during 2019 and 2020**


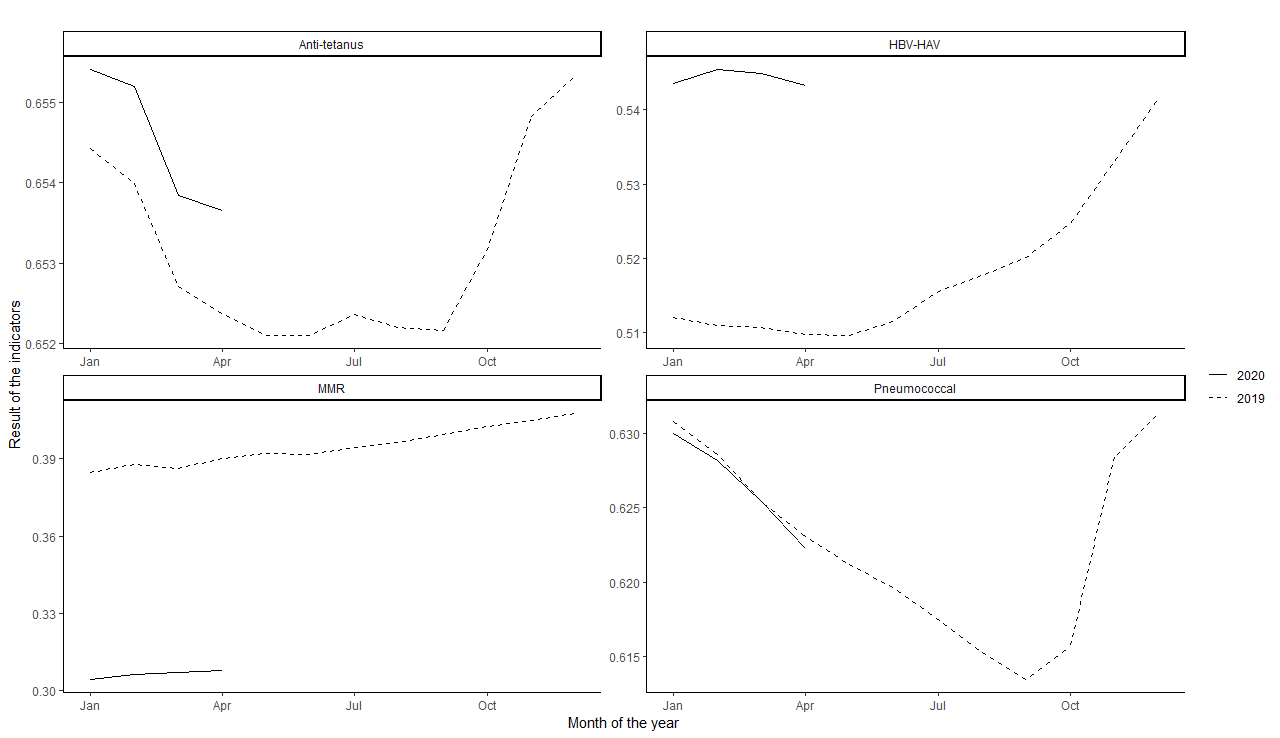


**Figure D Monthly result of EQA quaternary prevention indicators during 2019 and 2020
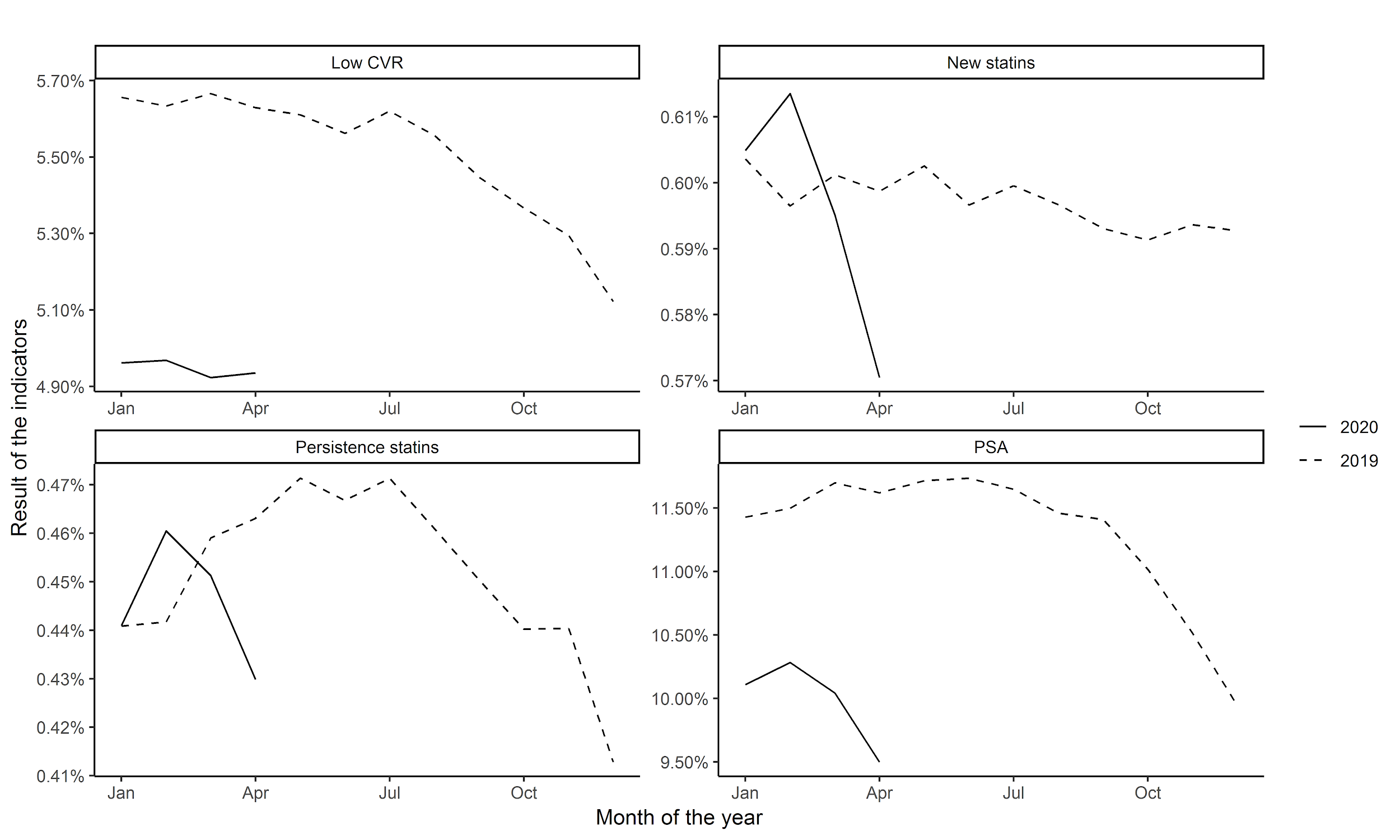
**
